# Paraphilia in Geriatric Patients: A Case Series From a General Hospital Setting

**DOI:** 10.7759/cureus.66260

**Published:** 2024-08-06

**Authors:** Surakshitha Poornima H. K., Debanjan Bhattacharjee, Tejaswi Prithviraj H. K.

**Affiliations:** 1 Psychiatry, Sri Chamundeshwari Medical College, Hospital and Research Institute, Channapatna, IND; 2 Psychiatry, Central Hospital Dhori, Phusro, IND; 3 Psychiatry, Adichunchanagiri Institute of Medical Sciences, Mandya, IND

**Keywords:** sexual sadism, exhibitionism, fetishism, sexual masochism, transvestic fetishism, zoophilia, stroke, paraphilia

## Abstract

Stroke can lead to various late-presenting complications that manifest weeks to months after acute stroke. While sexual dysfunction is common among stroke patients, hypersexuality and paraphilia are rare manifestations. This case series presents five cases of paraphilia showing the onset of abnormal sexual behaviors following an incident of stroke. The paraphilias in these five cases include sexual sadism, exhibitionism, transvestic fetishism, sexual masochism, fetishism, and zoophilia. Each case presents a unique manifestation of atypical sexual tendencies along with neuroimaging data and treatment approach. This case series contributes to the knowledge about the relationship between the incidence of stroke and the onset of paraphilia.

## Introduction

Stroke results in various adverse consequences that significantly affect an individual’s morbidity and quality of life. While the acute consequences of stroke are well recognized and adequately managed, the late-presenting medical complications are understudied [1,2]. Medical, musculoskeletal, and psychosocial complications may arise weeks or months after the acute stroke [2]. The psychosocial consequences of stroke that include poststroke depression, emotional lability (pseudobulbar affect), and mood/emotional changes negatively affect the patient, their family members, and friends [2].

The Diagnostic and Statistical Manual of Mental Disorders 5 (DSM-5) defines paraphilia as “an intense and persistent sexual interest other than sexual interest in genital stimulation or preparatory fondling with phenotypically normal, physically mature, consenting human partners” [3]. Paraphilic inclinations typically arise during adolescence [4]. In geriatric patients, they occur in the context of a neurological condition [5,6]. To the best of our knowledge, paraphilias have not been reported after stroke. This case series describes five such cases of new-onset paraphilia observed among geriatric patients following an incident of stroke.

## Case presentation

**Figure 1:**
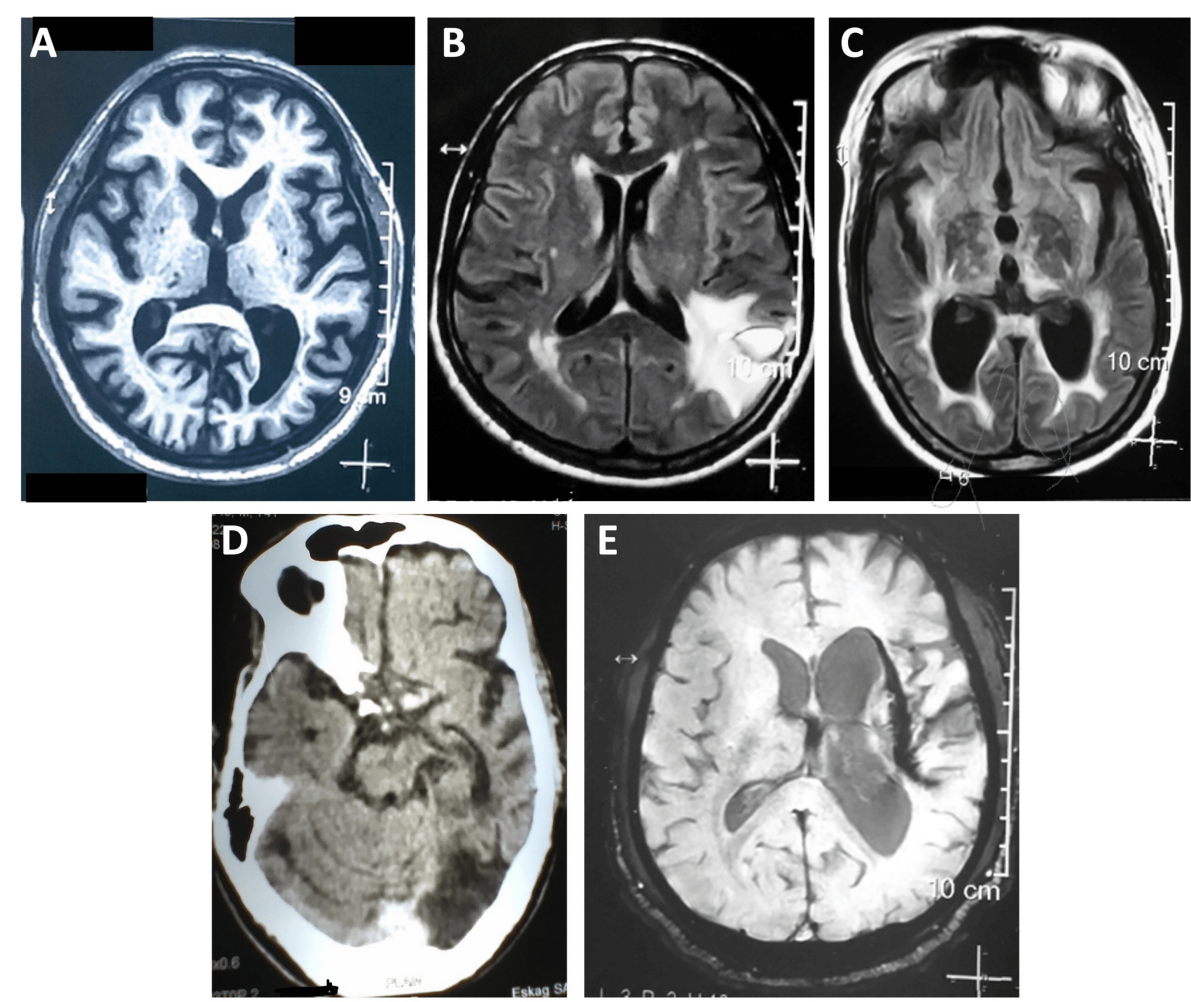
Noncontrast computed tomography images. (A) Patient 1 brain showing white matter ischemic changes in subcortical, periventricular, and bilateral basal ganglia region; microbleeds in the right frontal lobe, right gangliocapsular region, and right thalamus; and diffuse cortical atrophy in the midbrain. (B) Patient 2 brain showing hemorrhagic infarct with perilesional edema in the left posterior parietal lobe and periventricular and subcortical white matter ischemic changes. (C) Patient 3 brain showing normal pressure hydrocephalus, lacunar infarct in the left basal ganglia, and periventricular white matter ischemic changes. (D) Patient 4 brain showing acute ischemic infarct in the left cerebellum and left occipital lobe. (E) Patient 5 brain showing slit-like hemosiderin staining in the left gangliocapsular region with volume loss of the left cerebral hemisphere and ex vacuo dilatation of the left lateral ventricle and white matter ischemic changes

All patients had new-onset paraphilia following the stroke. None of the patients presented with a family history or any history of mood, psychotic, or anxiety disorders, or substance use. Figure 1 shows the neuroimaging results of each patient. Endocrinology test results evaluated by the respective physicians for all patients were within normal limits.

Case 1

A 72-year-old married male presented with symptoms of sexual sadism, involving recurrent and intense sexual arousal triggered by inflicting pain on his spouse. The symptoms had an insidious onset and an episodic course for the past 1.5 years. The patient’s medical history included an incident of stroke that occurred three months before the presentation of paraphilia. His medical records also mentioned stage 5 Alzheimer’s disease with major neurocognitive impairment. He was diagnosed with Alzheimer’s disease eight years back. Neuroimaging revealed multiple changes, including white matter ischemic changes located in subcortical, periventricular, and bilateral basal ganglia regions; microbleeds in the right frontal lobe, right gangliocapsular region, and right thalamus; and diffuse cortical atrophy in the midbrain (Figure 1A). He was treated with a trial of fluoxetine 60 mg and haloperidol 15 mg. Within six months of treatment, the patient showed substantial improvement in the symptoms of paraphilia.

Case 2

A 66-year-old married male presented with symptoms of zoophilia involving cattle and exhibitionism with recurrent and intense sexual arousal after engaging in sexual activity with cattle in the presence of other family members. The symptoms had an insidious onset and a continuous course for the past eight months. He had a history of hemorrhagic stroke five months before the presentation of paraphilia. Neuroimaging revealed a hemorrhagic infarct with perilesional edema in the left posterior parietal lobe (Figure 1B). He was treated with a trial of fluoxetine 60 mg and haloperidol 20 mg, which resulted in no improvement of the paraphilic symptoms. He was then switched to fluoxetine 60 mg and chlorpromazine 600 mg, wherein he showed partial improvement within four months of treatment.

Case 3

A 61-year-old married male presented with symptoms of sexual sadism characterized by recurrent and intense sexual urges from inflicting pain on his wife. The symptoms had an insidious onset and a continuous course for the past one year. He had suffered from an ischemic stroke three months before the presentation of paraphilia. He also had a medical history of dementia due to normal pressure hydrocephalus and was under treatment for the last three years. Neuroimaging revealed notable findings, including normal pressure hydrocephalus, a lacunar infarct in the left basal ganglia, and periventricular white matter ischemic changes (Figure 1C). He was treated with a trial of fluoxetine 40 mg and flupentixol 6 mg, which led to partial improvement in paraphilic symptoms over six months of follow-up.

Case 4

A 71-year-old married male presented with transvestic fetishism characterized by recurrent and intense sexual arousal by cross-dressing with feminine clothes and exhibitionism, displaying masturbatory behavior in the presence of family members. The symptoms had an insidious onset and a continuous course for the past six months. The patient had a history of ischemic stroke two months before the presentation of paraphilia. Neuroimaging revealed an acute ischemic infarct in the left cerebellum and occipital lobe (Figure 1D). He was treated with a trial of fluoxetine 60 mg and risperidone 2 mg, and his paraphilic symptoms substantially improved within three months of treatment.

Case 5

A 60-year-old married male presented with sexual sadism, sexual masochism, and fetishism involving recurrent and intense sexual arousal by inflicting pain on his wife and himself, often using vegetables for sexual activity. The paraphilia had an insidious onset and an episodic course for the past two years. The patient had a history of hemorrhagic stroke four months before the presentation of paraphilia. Neuroimaging revealed slit-like hemosiderin staining in the left gangliocapsular region with volume loss of the left cerebral hemisphere and ex vacuo dilatation of the left lateral ventricle, most likely a sequela of intraparenchymal hemorrhage, and white matter ischemic changes (Figure 1E). He was treated with a trial of fluoxetine 60 mg and chlorpromazine 600 mg, with partial improvement in symptoms after six months. He was then lost to follow-up.

## Discussion

This case series presents new-onset paraphilia in geriatric patients. All patients had an insidious onset of symptoms. While collating the medical history, we noticed that the onset of paraphilia was two to five months after an incident of stroke. Notably, all patients and their caregivers confirmed that there were no paraphilic inclinations before the incident of stroke. Additionally, patient 1 had moderately severe cognitive decline due to Alzheimer’s disease for the past eight years, and patient 3 has been treated for dementia for the past three years. None of the family members of the other patients reported any history suggestive of dementia. While dementia and neurocognitive decline may also contribute to the condition, we attribute stroke as the neurological insult responsible for the onset of paraphilic behaviors. Thus, paraphilia can be a delayed consequence of stroke.

Previously, multiple cases have been reported with paraphilia occurring in close association with brain disorders, including temporal lobe epilepsy, frontal lobe injury, Huntington’s disease, and frontotemporal dementia [7]. A case study reported the onset of paraphilic behavior following lesions in the right sides of the hypothalamus and mesencephalon and extending into the right sides of the red nucleus, substantia nigra, and internal capsule due to multiple sclerosis [8]. Paraphilia has also been reported in the context of Parkinson’s disease [5]. However, we have not come across literature showing the onset of paraphilic behaviors following a stroke.

Multiple brain areas have been implicated in paraphilia, including the dorsolateral and orbitofrontal cortex, temporal cortex, temporoparietal cortex, amygdala, hippocampus, frontostriatal circuits, and cerebellum [9,10]. Further, white-matter abnormalities in these areas and abnormal blood flow, including temporal hypometabolism, have been associated with an increase in impulsivity and sex drive [10]. Stroke can cause long-term neuroplastic changes, including gamma-aminobutyric acid (GABA) receptor activity in the affected and connected areas [11]. GABA dysfunction in the dorsal anterior cingulate cortex and its connections with the limbic system have been observed in paraphilia [10]. Temporal lobe disturbances, seen in dementia, can also be associated with the onset of paraphilic behaviors [6]. Additionally, dysregulated testosterone levels have been implicated in paraphilia [12]. Abnormal levels of the neurotransmitters serotonin, norepinephrine, and dopamine have been reported in patients with paraphilic disorders [13].

Paraphilia is a controversial topic [14]. The definition of normal and deviant sexual behavior depends on the society. The DSM-5 defines the paraphilic disorder as “a paraphilia that is currently causing distress or impairment to the individual or a paraphilia whose satisfaction has entailed personal harm, or risk of harm, to others” [3]. The distress caused by paraphilia and the occurrence of symptoms over a period of six months are a prerequisite for the diagnosis. Moreover, treatment is not warranted for paraphilia itself [3]. The patients from this case series showed paraphilic behavior for six months to two years before they presented to the psychiatric department. The reason for this delay can only be speculated. It could be because of the absence of distress, accommodation of the behavior, or sheer embarrassment. In this case series, we did not diagnose the patients with paraphilic disorder as per DSM-5. We refer to their behavior as paraphilia.

The families of the patients brought them to the psychiatric department to treat the paraphilic behavior. All the cases presented in this case series were treated with a combination of selective serotonin reuptake inhibitors and first-generation antipsychotics with mixed results. This approach is supported by the treatment guidelines of the World Federation of Societies of Biological Psychiatry (WFSBP) for paraphilic disorders [15]. Notably, specific guidelines for the treatment of paraphilia in geriatric patients are lacking. The WFSBP 2020 guidelines also recommend hormonal treatment for patients with paraphilic disorders [15]; however, patients and caregivers did not consent to hormonal treatment, restricting its use in our cases.

While this case series presents the rare phenomenon of paraphilia secondary to stroke in geriatric patients, more cases with detailed neuroimaging are required to draw specific conclusions about structural brain areas implicated in paraphilia. More case studies will also provide information to devise better treatment strategies for new-onset paraphilia in geriatric patients.

## Conclusions

This case series highlights that new-onset paraphilia can occur in geriatric patients following a neurological insult due to stroke. It can be a delayed consequence of stroke. Neurocognitive changes in geriatric patients may also contribute to the paraphilic inclination. Further studies are required to understand the neuroanatomical basis of paraphilia and formulate clinical practice guidelines for late-onset paraphilia that may occur secondary to a neurological insult.
